# An extremely simple macroscale electronic skin realized by deep machine learning

**DOI:** 10.1038/s41598-017-11663-6

**Published:** 2017-09-11

**Authors:** Kee-Sun Sohn, Jiyong Chung, Min-Young Cho, Suman Timilsina, Woon Bae Park, Myungho Pyo, Namsoo Shin, Keemin Sohn, Ji Sik Kim

**Affiliations:** 1 Faculty of Nanotechnology and Advanced Materials Engineering, Sejong University, Seoul, 143-747 Republic of Korea; 2 Laboratory of Big-data applications for public sector, Chung-Ang University, 221, Heukseok-dong, Dongjak-gu, Seoul, 156-756 Republic of Korea; 3 School of Nano & Advanced Materials Engineering, Kyungpook National University, Kyeongbuk, 742-711 Republic of Korea; 4 Department of Printed Electronics Engineering, Sunchon National University, Chonnam, 540-742 Republic of Korea; 5 Deep Solution Inc., 2636, Nambusunhwan-ro, Seocho-gu, Seoul, 06738 Republic of Korea

## Abstract

Complicated structures consisting of multi-layers with a multi-modal array of device components, *i.e*., so-called patterned multi-layers, and their corresponding circuit designs for signal readout and addressing are used to achieve a macroscale electronic skin (e-skin). In contrast to this common approach, we realized an extremely simple macroscale e-skin only by employing a single-layered piezoresistive MWCNT-PDMS composite film with neither nano-, micro-, nor macro-patterns. It is the deep machine learning that made it possible to let such a simple bulky material play the role of a smart sensory device. A deep neural network (DNN) enabled us to process electrical resistance change induced by applied pressure and thereby to instantaneously evaluate the pressure level and the exact position under pressure. The great potential of this revolutionary concept for the attainment of pressure-distribution sensing on a macroscale area could expand its use to not only e-skin applications but to other high-end applications such as touch panels, portable flexible keyboard, sign language interpreting globes, safety diagnosis of social infrastructures, and the diagnosis of motility and peristalsis disorders in the gastrointestinal tract.

## Introduction

There have been incessant attempts to produce artificial skin, so-called electronic skin (e-skin), which could approximate the function of actual human skin^1–8^. A key attribute of the artificial skin functions would be tactile sensing consisting of the recognition of both a touching position and strain (or pressure) exerted at that position. Although other sensing functions such as thermal and chemical sensing should also be accorded parallel status, we focused only on position and strain sensing in the present investigation.

Many brilliant strain sensors have recently been developed for use in e-skin applications^1–8^. No matter what types of strain sensors are used for e-skin, a clear common feature of all existing e-skins has been the orderly arrangement of device components on a macroscale area, which is a so-called pattern. These patterns of multi-modal arrays arranged in either single or multi layers, are inevitable in what can be considered e-skin. Otherwise, there would be no possible way to achieve a systematic detection of the pressure distribution on a macroscale area of skin.

In this regard, a number of tactile or position sensory devices are well established: resistive, capacitive, Inductive, piezoresistive, optical, magnetic, binary, piezoelectric, and hydraulic^9–12^. All of these are composed of a pattern of device components on multi-layered substrates along with a logic circuit design for signal readout and addressing. There are a variety of pattern types for e-skin, from the simplest of spacer patterns between layers for a resistive touch panel to extremely complicated wire arrays for strain-gauge sensors and even TFT array patterns. The pattern preparation methods range from ink-jet or roll-to-roll printing to a complicated semiconductor process. Complicated manufacturing processes for sensing devices, however, is one of the most serious barriers to the development of promising e-skins at a low cost.

We completely changed the paradigm of strain sensing (and position) by suggesting a novel strategy that precludes nano-, micro-, and macro-device patterns, and thereby achieved a tremendously low-cost e-skin. Accordingly, neither orderly patterned nor multi-layered structures were required, and a brilliant strain (and position) sensor covering a large area was realized by adopting a macroscale piezoresistive composite sheet that consisted of homogeneously dispersed multi-wall carbon nanotube (MWCNT) in a polydimethylsiloxane (PDMS) matrix. Although CNT-related materials have been utilized as nano-, micro-, and macro-device components for existing e-skins^3, 13, 14^, large, intact, crude MWCNT-PDMS composite sheets are considered obsolete. In the present study, however, we re-introduced this simple bulky material, and it was reborn as an extremely smart sensor with no need for complicated device fabrication process. In other words, such an extremely simple macroscale material could replace all of the complicated device structures that consist of multi-layered patterns.

Deep machine learning made it possible for such a simple bulky material to play the role of a smart sensory device, and it also enabled the use of a pattern-free pressure (and position) sensor for use in artificial skin and other applications. The electrical signals detected from several arbitrary points on the edge of a flat MWCNT-PDMS composite sheet were processed via a deep neural network (DNN), so that the touch point recognition as well as the exact pressure sensing could be realized. The key to this novel e-skin is that a simple material can replace complex patterned devices via the assistance of a DNN. Although a similar approach to pattern-free strain sensor (Piezoresistive MWCNT-silicone rubber composite) based on the electrical impedance tomography (EIT) has been just reported^15^, it is obvious that the DNN-driven sensor conspicuously outperforms the EIT-driven one in terms of pressure sensitivity and spatial resolution.

The recent advent of deep learning techniques has given scientists and engineers a new point of view for a variety of traditional scientific and engineering issues. Modeling that is based on a DNN is generally referred to as deep learning. Artificial neural networks (ANNs) with a single hidden layer were developed in the 1940s and continued to prosper throughout the 80 s and 90 s^16–23^. During the same period, ANNs were applied to a variety of tasks. Unfortunately, ANNs with a shallow structure were nearly abandoned after the new millennium, since their performance did not exceed that produced from conventional rule-based engineering. The term “deep-learning” was born from the failure of previous shallow-learning structures with a single hidden layer. DNNs were created by simply employing multiple hidden layers in an ANN.

In the initial stages of developing training methods for DNNs, many researchers suffered from an identification problem due to the need to estimate so many weight parameters. Training a DNN with small-sample data definitely led to the fatal problem of over-fitting. Hinton *et al*.^24^ proposed a robust way to circumvent the problem. They suggested a pre-training model with unlabeled data, which provided an initial solution for weight parameters. Stacking multiple restricted Boltzmann machines (RBMs) made it possible to provide a plausible initial solution for a DNN. This pre-training method clearly triggered a boost in deep learning. However, recent efforts to secure labeled big data and improvements in processing speed are more responsible for enhancing the performance of DNNs^25^. Deng and Yu^26^ found that carefully designed random initial parameters could resolve the over-fitting problem without the need for pre-training, but only if a large amount of training data are available. In this regard, we established a deep architecture in the development of this novel e-skin using big data that incorporated 540,000 input vectors.

## Basic concept of the proposed approach

**Figure 1:**
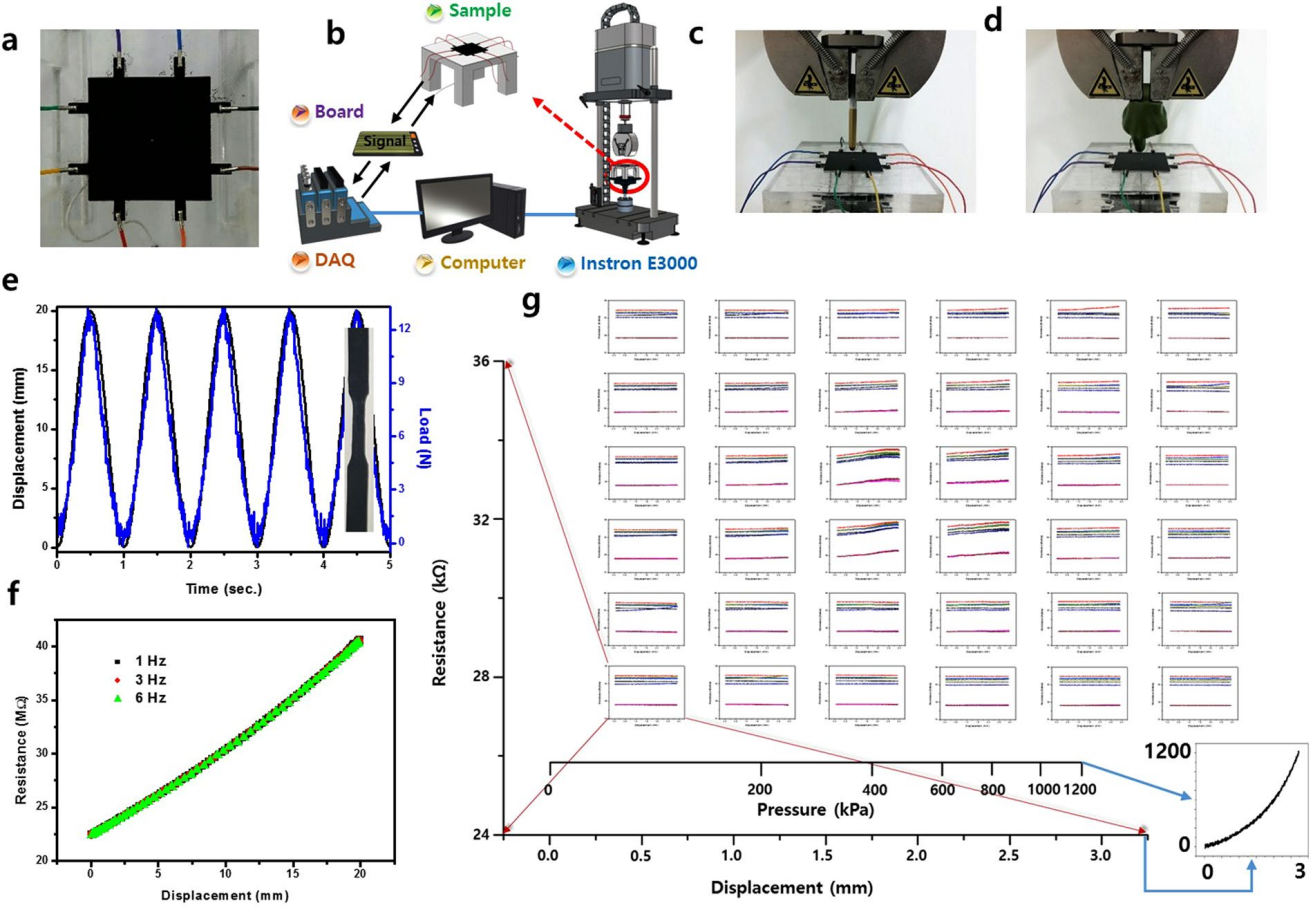
The e-skin and its details for training and test. (**a**) MWCNT-PDMS sheet acting as an e-skin (40 mm × 40 mm). (**b**) Piezoresistive resistance measurement set up. (**c**) The wooden bar used for training. (**d**) A toy finger used for test. (**e**) A plot of load (displacement or pressure) and electrical resistance as a function of time for a cyclic loading with a frequency of 1 Hz for a uniaxial tensile specimen as given in the right side of the plot. (**f**) The electrical resistance versus displacement plot for the uniaxial tensile specimen at frequencies of 1, 3, and 6 Hz, showing a complete coincidence. (**g**) 8 probe terminal electrode signals versus the displacement (pressure) at 6 × 6 sector positions, and a pressure versus displacement curve in the range of pressure from 0 to 1.2 MPa (right side on the bottom).

The key function for artificial skin is an instantaneous recognition of strain (pressure) distribution on a macro-scale area in response to mechanical stimulus. More simply, it would be the recognition of both the pressure and the position under pressure. An orderly pattern of device components (=sensor matrix) on a macro-scale area is required to realize such a function. However, the proposed novel e-skin involves no such pattern, and, instead, is a flat MWCNT-PDMS sheet, which is nothing but homogeneously dispersed piezoresistive composite material. Figure 1(a) shows an actual photo of the specimen in detail, exhibiting no patterned structure on the top surface. The bare sheet area was hypothetically divided into m × m small sectors (m = 4, 6, and 10). This virtual lattice type of compartmentalization allowed a single sector area (=spatial resolution) to approximate the size of a fingertip contact. The resistance changes instantaneously detected at n-probe terminals (n = 4, 8, and 16) were recorded with respect to repetitive pushing on each sector in turn. The final goal of the present machine learning was to achieve an exact DNN model of instantaneous touchpoint recognition and to simultaneously evaluate the pressure level by reading the resistance signals measured at the 4-, 8-, or 16-probe terminals located at the edge of the e-skin.

First, we constructed a DNN for position recognition. The input data were vectorized as n-dimensional vectors representing each of the electric resistances measured at the n-probe terminals (n = 4, 8, and 16), and the output data were vectorized as m^2^-dimensional label vectors representing the m × m divided sectors (m = 4, 6, and 10). A wooden stick was used to generate the training dataset, but the test dataset was separately prepared using a plastic toy finger. Along with the optimum DNN architecture construction, the probe terminal arrangement for electrical resistance measurement was designed such that we were able to achieve an acceptable trade-off between the number of probe terminals and the reliability of the learning results. That is, the number of probe terminals should be minimized while the test accuracy of the DNN should be maximized. The minimum numbers of probe terminals needed to guarantee a clear resolution of the electrical resistance data leading to acceptable levels of test accuracy for a DNN were 8 and 16, which resulted in a perfect learning result for all virtual lattice compartmentalization schemes (m = 4, 6, and 10). Table 1 clearly exhibits the position recognition test accuracy for the 4-, 8-, and 16-probe terminals for the 6 × 6 lattice, and shows that 4-probe terminals did not result in a high level of accuracy.

**Table 1:** All CNT-PDMS sheets are virtually segmented into 6 × 6 sectors (with spatial resolution of 6.6 mm).

All CNT-PDMS sheets are virtually segmented into 6 X 6 sectors (with spatial resolution of 6.6 mm)	Position Recognition	DNN Architecture (The number of nodes at each layer)	
		Training Accuracy Individual data point basis (%)	Test Accuracy Individual data point basis (%)
		Training Accuracy Group data basis (%)	Test Accuracy Group data basis (%)
	Pressure Sensing	DNN Architecture (The number of nodes at each layer)	
		%RMSE	MAE
16 probe terminal electrodes (16 features)	Position Recognition	16-1024-256-64-36	
		99.96	99.28
		100	100
	Pressure Sensing	16-4096-1024-128-1	
		3.96	0.053
8 probe terminal electrodes (8 features)	Position Recognition	8-5760-1152-144-36	
		98.97 (89.16)	96.19 (85.54)
		100 (99.72)	97.22 (100)
	Pressure Sensing	8-2048-256-1	
		3.12 (3.49)	0.045 (0.030)
4 probe terminal electrodes (4 features)	Position Recognition	4-2304-576-144-36	
		87.87	51.32
		99.17	50
	Pressure Sensing	N/A	

The DNN architectures and the training/test accuracies for position recognition and pressure sensing (0~1.2 MPa) for three MWCNT-PDMS sheets with different probe terminal structures. The figures inside the parenthesis are the thin, soft sheet data, which represent the tactile pressure range (0~70 kPa). More details about the DNN for the thin, soft sheet are presented in the Supplementary information (S8).

When comparing the present novel tactile sensing system with either the conventional resistive or capacitive sheet that is known as the simplest touch panel system^27^, the present version was superior to the resistive sheet in terms of simplicity. A resistive touch panel consists of two sheets divided by an orderly spacer pattern, and the projected capacitive touch panel consists of a large number of electrodes in a pattern. In contrast to the resistive and projected capacitive touch panels, however, the MWCNT-PDMS has a single-layered (single sheet) structure with no orderly pattern. More importantly, it would be unreasonable to compare the MWCNT-PDMS sheet designed for e-skin with such a simple touch panel since the MWCNT-PDMS sheet made it possible to sense both the position and strain simultaneously, but the touch panel allows for position sensing alone. Compared with other more complex devices, the MWCNT-PDMS sheet was superior in terms of structural simplicity.

## Artificial Neural Network Architecture

**Figure 2:**
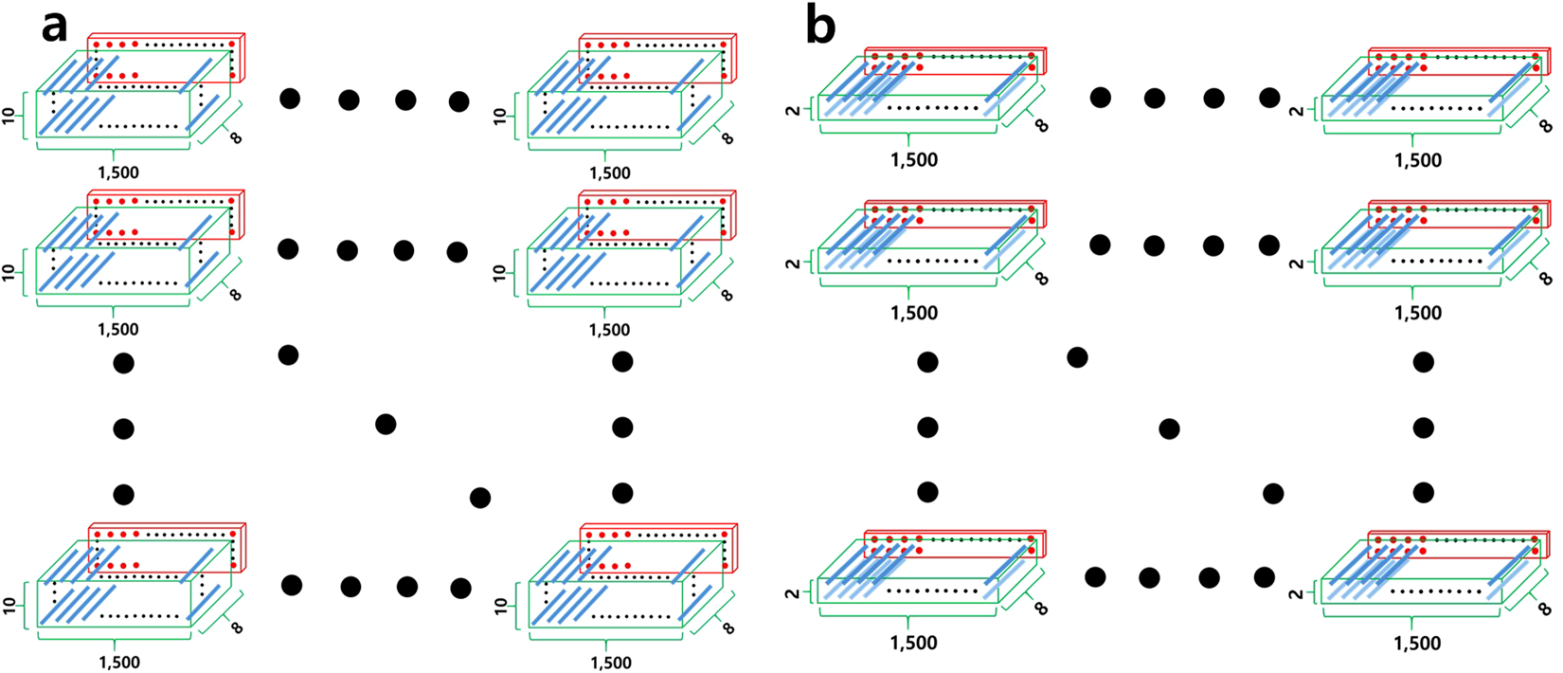
The dataset structure. (**a**) Training dataset structure consists of a green box and a red slab; green box represents input data involving 15,000 (=1,500 × 10) 8-dimensional vectors (blue bars), the 8 resistance values constituting each vector were instantaneously measured from 8 probe terminals connected to the CNT-PPDMS sheet, and red slab denotes displacement (pressure) matrix, wherein red dots are pressure values corresponding each input vector. The total dataset consists of 36 (6 × 6) sectoral datasets and thereby 540,000 resistance input vectors and pressure values were used for the DNN training. (**b**) The test dataset also has the same dataset structure but not the same shape and dimension exhibiting a green slab and a red thinner slab, each of which represents 3,000 (=1,500 × 2) 8-dimensional vectors (blue bars) and the corresponding pressure values, respectively. Note that the most important point here is that the training dataset was made by a wooden bar but the test dataset was made by a toy finger.

Basically our DNN architecture consists of two channels for position recognition and strain (pressure) recognition. These two channels operate simultaneously in actual sensing situations. The input feature was vectorized into n-dimensional (1 × n) vectors, the component of which denotes a real resistance number measured from each of the n-probe terminal electrodes (n = 4, 8, and 16). A certain level of pressure (from zero to 1.2 MPa) was applied on a sector of concern for a second and data were collected while pressure was applied. A schematic diagram of the whole experimental setup for loading is shown in Fig. 1(b). As a result, we collected 1,500 n-dimensional input vectors along with the corresponding 1,500 pressure values per each sector, and 10 independent measurements were implemented at each sector, so that the total training dataset consisted of 540,000 (1,500 × 36 × 10) n-dimensional input vectors for the 6 × 6 lattice. The training and test dataset structure for m = 6 is schematically described in Fig. 2. The label (output) data for position recognition was simply a sector coordinate that designated the column and row numbers of the 6 × 6 matrix, and the label data for displacement (pressure) was a real number. The test dataset had a similar data structure but smaller than the training dataset, and was separately prepared by a toy finger that differed from the wooden bar used for the training dataset. Figure 1(c) and (d) show the wooden bar and the plastic toy finger for use in training and test, respectively.

The piezoresistive resistance of MWCNT-PDMS material is known to be strain-rate independent^28^. This means that the piezoresistive resistance is only dependent on the instantaneous state of the conductive CNT distribution in an insulating PDMS matrix, but not on the strain rate. The piezoresistive resistance exhibited a complete linear relationship with the strain (displacement or pressure) irrespective of the loading frequency, as shown in Fig. 1(e–f). Thus, the expression “pressure” can be used along with “strain” and “displacement.” In fact, the terms “pressure,” “displacement,” and “strain” should be treated equally as output signals with no distinction among them from a practical point of view, since they all exist in completely elastic relationships both in static and in dynamic conditions, and show neither anelastic nor plastic behaviors. Consequently, it makes no difference which term is used in this instance.

The DNN architecture was classified according to the number of hidden (intermediate) layers, the number of nodes (neurons) at each layer, the details of activation functions, dropout, batch size, epoch control, and validation set setting. The number of nodes at the input layer is equal to the number of features, the input vector dimension, and the number of probe terminal electrodes. The final architecture was determined through many trials from scratch. The training and test results for only the DNN architecture with 8 features (8 input nodes) and 36 (6 × 6) labels (﻿36 output nodes) are described in detail in the next subsection, and the details of the other DNN architectures with 16 and 4 features are presented in the Supplementary information (S1). The versions of DNN architecture along with their training and testing results, are summarized in Table 1, and the schematic representation of DNN architectures with 4, 8, and 16 features are presented in Figure S1. A slightly higher accuracy for training and test was obtained in the case of 16 features. However, for the sake of simplicity fewer features (fewer number of probe terminal electrodes) would be more favorable, so that an e-skin device involving 8-probe terminal electrodes would be more desirable than one with 16 electrodes. In the case of the 4-probe terminal electrodes, the training and testing accuracy deteriorated dramatically, which did not allow position recognition that in turn made pressure sensing impossible. A trade-off between the electrode number and accuracy occurred at the number 8 in our pattern-free e-skin on a 16 cm^2^ area.

In fact, we produced another dataset for a DNN with 4, 8, and 16 features and 16 labels (i.e., 4 × 4 lattice case). Although the accuracy of the training and test was much better than that for the 6 × 6 lattice, the training and test results from these data were omitted. Since a finer sector division was more desirable for a more sophisticated tactile sensor, the data from the 6 × 6 lattice would be more realistically simulate the actual skin. Partial results for the 4 × 4 division sheet are available in the Supplementary information (S2). In addition, an e-skin with 10 × 10 lattice (100 labels) exhibiting a tremendously improved spatial resolution for pressure sensing is well described in the Supplementary information (S3).

### Touch position Recognition DNN with 8 features and 36 labels

**Figure 3:**
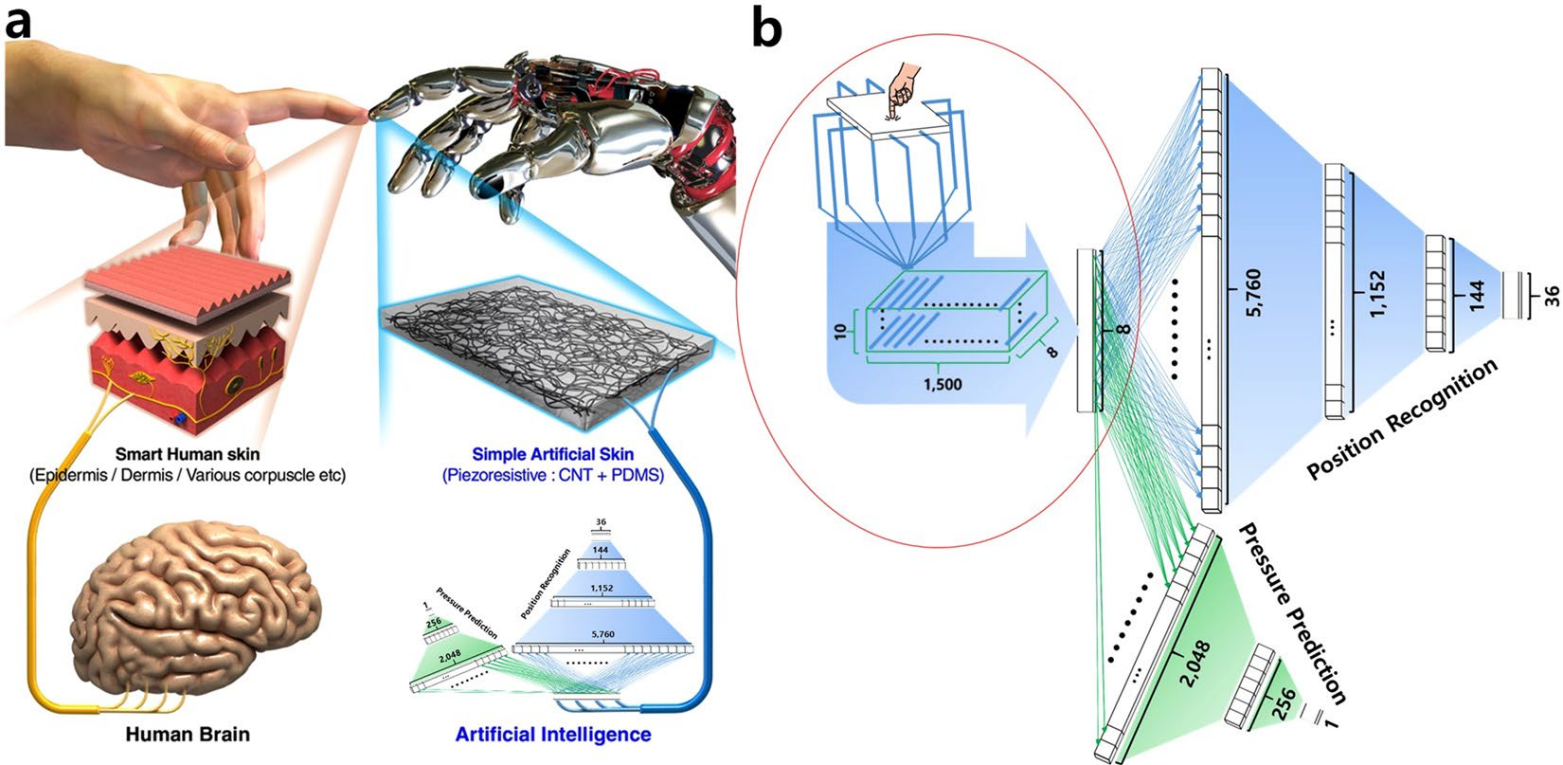
The basic concept for e-skin and DNN architecture for reliable sensing. (**a**) A schematic elucidating the comparison between the human skin and the suggested e-skin made of an extremely simple, crude, and macroscale MWCNT-PDMS composite sheet with neither nano-, micro-, nor macro-patterns. (**b**) The DNN architecture for tactile sensing. A schematic of MWCNT-PDMS sheet was depicted showing 8 probe terminals along with a sectoral dataset structure is also presented inside a red circle. The two channel DNNs are given, the sky blue colored DNN architecture is designed for the position recognition (upper) and the greenish one represents the pressure evaluation model (bottom).

We focuses on a DNN with 8 features and 36 labels as a model case in the present investigation. The electrical resistance signals collected from 8-probe terminal electrodes were featured as 8-dimension input vectors in the DNN for position recognition. The DNN architecture for position recognition is composed of three fully connected hidden layers, as shown in Fig. 3. There are 8 nodes in the input layer, 5,760 nodes in the first hidden layer, 1,152 nodes in the second hidden layer, 144 nodes in the third hidden layer, and 36 nodes in the output layer (architecture: 8; 5,760; 1,152; 144; and, 36). The rectified linear unit (ReLu), which is max(0, x) with a threshold at zero, was adopted as an elemental activation function for the three hidden layers, and a 30% dropout was introduced. The activation function for the last fully connected layer was a softmax function. Validation was implemented every 100 epochs at a rate of 0.25.

The training accuracy was 98.97% on an individual training data basis. The training accuracy reached 100% when all data corresponding to a particular touch were bound together in a group for a particular block (sector), and thereby a dataset composed of 1,500 input vectors with 1,500 labels was considered as a group representing each independent touch. Each touch generated a dataset (1,500 input vectors) that in principle should designate the corresponding label (=the sector that were under pressure). However, some of the 1,500 input vectors were mislabeled, but the number was negligible. The most frequently matching label was regarded as a correct sector indicated by this group data. A full measure (100%) of accuracy was obtained by a complete match between the most frequent label and the actual label, which showed that the DNN model hit the label of 360 training dataset groups without a single failure. Every touch was successfully recognized by DNN since 360 training dataset groups indicate 360 touches.

Common knowledge suggests a certain portion of training data should be separated and used to test a conventional DNN setup. However, we did not follow this convention, and, instead, we prepared separate test datasets using a completely different pressing bar (toy finger) for the purpose of securing a robust DNN. The test dataset size was smaller than the training dataset, which involved 108,000 (1500 × 36 × 2) input vectors with labels for the 6 × 6 lattice. The test was also executed on both an individual data basis and a group data basis. Of course, we implemented conventional training and test by setting aside 20% of the training dataset for test, and the resultant accuracy was 99.78% for individual data and 100% for group data in this conventional test. This means that from instantaneously detected 8 electrical resistance values, an exact touch point can be picked at every single moment with a time resolution of ~10^−4^ sec. with an accuracy of 99.78%. Also the group-data-based test accuracy in this conventional case reached 100%.

The individual data-based test accuracy with the toy finger was obtained to be 96.19%, and the test accuracy for the group data basis with the toy finger ultimately reached 97.22%. The group-data-based test accuracy stemmed from the testing of 72 arbitrary touches. A group-data-based test accuracy of 97.22% for the position recognition denotes that only two out of the 72 datasets were missed, indicating that only a two touches were misled by DNN out of the 72 touches. It should be noted, however, that even these two misled recognitions were not far from the correct position, and were in a sector adjacent to the correct sector under pressure. This sort of error might happen even with actual skin since the spatial resolution of the proposed e-skin was around 6.6 mm, which is far smaller than the fingertip contact area.

It would be futile to discuss such a small error for the touch position recognition since it is obvious that we definitely obtained 100% accuracy in the test for the 16-probe terminal electrodes, as shown in Table 1. The individual-data-based test accuracy is 99.28 and the test accuracy for the group data basis ultimately reached 100%. This means that every touch was correctly recognized by the 16-feature DNN model. However, the 8-feature DNN model also worked properly and the performance was acceptable and we focused more on it for the sake of a device brevity. An extremely high spatial resolution was obtained for a 10 × 10 segmented e-skin (16 features) with a group-data-based test accuracy of 100%. Furthermore, even the real number of spatial coordinates (x and y) were also successfully modeled with a resolution of 0.78 ± 0.44 mm by a regression-typed DNN. These results are presented in detail along with reasonable comparisons with the existing sensors in the Supplementary information (S4, S5, and S6).

### Pressure Evaluation DNN with 8 features and 36 labels

Other types of DNNs were separately built up for the strain (pressure) evaluation, as shown in Fig. 3, wherein the architecture consisted of 8-2048-256-1 nodes belonging to each layer. Only datasets belonging to a particular sector under pressure (touched sector) were used for the pressure evaluation since the position recognition was readily completed by the primary DNN channel. Accordingly, the DNN for pressure evaluation was trained and tested by employing 15,000 input vectors with the corresponding pressure values per each sector and a total of 540,000 (15,000 × 36) input vectors, as shown in Fig. 2. ReLu was adopted as an activation function for hidden layers as in the position recognition DNN. However, the activation function of the last layer of the DNN for the pressure evaluation was not a softmax, but was instead a plain linear function, so that the final output value was a real number that designated the displacement, which was then regarded as either strain or pressure according to the elastic relationship that existed.

**Figure 4:**
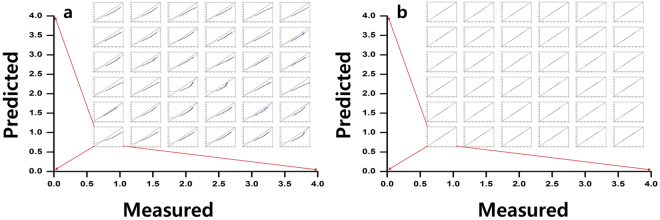
The pressure evaluation process for 6 × 6 sectors. (**a**) Predicted vs measured displacement (pressure) curves for 1,000 test data points (primary test dataset) for each of the 36 sectors. The diagonal straight line in each graph stands for the optimal data status but the actual result deviated significantly from it. The red line shows the result from the polynomial function regression to model the deviation. (**b**) Predicted vs measured displacement for the other 500 test data points (confirmative test dataset) after the data transformation based on the polynomial regression result, showing the perfect coincidence between the predicted and measured displacement for every sector. This result shows a high precision pressure sensing with brilliant fitting parameters such as %RMSE (3.12) and MAE (0.03).

The test of the pressure evaluation DNN was also implemented using the toy finger data, which amounted to 1,500 input vectors per sector and totally 54,000 input vectors. We randomly divided the 1,500 input vectors into 1,000 as the primary test dataset and 500 as the confirmative test dataset. When the 1,000 primary input vectors and corresponding pressure values were used for the DNN test, the test accuracy was not acceptable, and a series of mismatches were shown between the predicted and the experimental values. Figure 4(a) shows the plots of predicted versus experimentally measured displacement (pressure) for each of the 36 sectors. The predicted pressure deviated significantly from the experimental data, and exhibited a formation of non-linear curves that departed from the diagonal line, as shown in Fig. 4(a). Such a deviation was simply a biased (transformed) feature rather than a scattered type. This means that a correction could be available via simple data transformation. In this regard, we introduced a regression by introducing a second-order polynomial function as a regression model (In(y) = *β*_0_ + *β*_1_ *x* + *β*_2_ *x* ^2^), and the regression result is depicted by the red line in Fig. 4(a). Then, we tested the DNN by employing a confirmative test dataset consisting of 500 input vectors and output pressure values per each sector and incorporated the data transformation that resulted from the polynomial regression. Finally, we achieved a reliable DNN model for the pressure recognition based on the DNN and the ensuing non-linear regression. Figure 4(b) shows the confirmative test dataset, exhibiting plots of predicted versus experimental pressure for each of the 36 sectors. The DNN modeling and the ensuing regression transformed the confirmative test dataset, and a complete coincidence was achieved between the predicted and the experimentally measured pressures. Such a computational process enabled us to immediately predict a pressure level with high precision for a position under pressure. The pressure sensing was satisfactory, since the %RMSE and MAE averaged over 36 sectors was estimated to be 3.12 and 0.03, respectively. These values would be thoroughly acceptable when the DNN is applied to actual artificial skin.

### Spatial resolution and pressure sensitivity

The spatial resolution of pressure sensing for the suggested e-skin advances the present capabilities of existing sensors. More importantly, it is extremely easy to improve the spatial resolution with no need to physically alter the device structure. It is possible to achieve better resolution by simply changing the training condition by varying the number of features (the number of probe terminals), the virtual areal segmentation scheme, and the amount of data. We obtained a spatial resolution of 6.6 mm for the e-skin with a 6 × 6 virtual lattice. The spatial resolution can be further reduced by adopting a finer virtual lattice and by increasing the number of probe terminals. It is unnecessary to change the e-skin structure to improve the spatial resolution. For example, we reduced the spatial resolution down to around 4 mm, when the e-skin sheet with a size of 40 mm × 40 mm was trained by adopting 10 × 10 virtual lattice and 16 probe terminals. The spatial resolution of the most advanced tactile sensor is around 2.5 mm^1–3^. The degree of freedom for virtual segmentation designs is infinite in principle, so that we can achieve whatever resolution could be. However, the above-described pick-block type test was in reality restricted to a limited choice of resolutions because of the time and cost issues. To overcome this limitation, a regression-typed DNN was introduced and the real number of spatial coordinates (x and y) were successfully modeled with a resolution of 0.78 ± 0.44 mm. Surprisingly, the real value-based regression DNN allowed for such a tremendously enhanced spatial resolution that conventional patterned device cannot catch up with. More details on the regression-typed DNN are presented in the Supplementary information (S4, S5, and S6).

Since the DNN model elicited a single pressure value from 8 electrical resistance signals, it is much more advantageous to secure higher pressure sensitivity in a fast time frame (<10^−4^ sec.) than is possible with a conventional pressure sensing system, wherein a pressure value is converted from a single resistance value. For a macroscale e-skin consisting of a patterned sensor matrix, a single sensor in charge of pressure sensing at a specific location is actuated to evaluate the pressure value at the location. Pressure values at the location are predicted from a single pressure vs. resistance curve based on a one-to-one relationship. Eight-to-one (or sixteen-to-one) derivations for pressure sensing in the DNN-driven e-skin would provide much more sensitive pressure sensing than one-to-one derivations, especially when using highly noisy data with a small gradient. Figure S9 in the Supplementary information (S7) shows pressure vs. resistance plots exhibiting eight-to-one relationships for a thick (hard) sheet and for a thin (soft) film. All the pressure vs. resistance curves in Figure S9 exhibited sufficient gradients with very low noise levels providing a reliable pressure sensitivity. However, the DNN-driven sensor can work in a much worse case.

## Discussion

It is curious as to how a DNN is so capable of sorting out complex problems. Our understanding is that a DNN cannot perform a task that humans could not—if given a sufficient amount of time. For instance, picture recognition was recently one of the hottest convolutional neural network (CNN)-related issues, and is an easy task for human beings in everyday life. However, a CNN can perform picture recognition much faster than humans. A CNN can classify a tremendously large number of pictures in a much shorter amount of time. In the same context, a human cannot simultaneously monitor the 8 or 16 resistance curves as per each touch on a certain sector for a second, but a DNN can. If we kept scrutinizing 288 or 576 resistance curves for 36 sectors for several hours, then we would notice a slight difference in a tiny part of these data and it would be possible to achieve the position recognition. However, a human would never notice anything in a real timeframe, but the DNN timeframe is much faster.

In fact, it is impossible to derive a rule-based model that can replace a DNN. The quantity and quality of data play a crucial role in achieving a reliable DNN model. In particular, It is evident that high-quality data make the largest contribution to success^29, 30^. The proposed e-skin operates through a so-called data-driven approach, wherein we must read what the raw data implicate, and the DNN model just acts as a medium to extract information from the data. In addition, we utilized no feature engineering but fed raw data directly to the DNN. In the same context, the present study depends on neither predefined rules nor human intervention.

The e-skin prototype has proven to work properly with high precision both in terms of position recognition and pressure evaluation. It should be noted, however, that the e-skin prototype that we developed in the present investigation is far from complete by comparison with real human skin. A simple push on a specified sector area, which we adopted for the training of the DNN, would be an extremely tiny part of all instances of stimuli that the human skin can experience. However, a DNN could work in much more complex pressure application cases such as shearing, friction, rubbing, and any of the more complicated types of pressure applications even on larger and irregular-shaped areas. Such complications can be sorted out by the DNN rather than by device improvement. Namely, if a sufficient amount of training data and a sufficient computation capacity for the training could be secured, nothing would be impossible. In this regard, the instantaneous recognition of a real distribution of pressure upon a complicated stimulus would be available, no matter the complexity of the skin shape or the stimulus type.

The versatility and robustness of the DNN-assisted e-skin was validated by switching the pressing bars for training and testing, whereby the DNN training data were gathered using a wooden bar but the test data were collected using a plastic toy finger, as shown in Fig. 1(c) and (d). The shape of the contact point, the hardness, and the elastic constant all differed sharply. However, we confirmed that the test accuracy was as close as 100%. We actually used the test dataset produced by the same wooden bar, and the test accuracy was improved regardless of the DNN architecture. In fact, although the resistance and pressure profile precisely differed between the wooden bar and the toy finger, the overall trend looked similar.

One source of misunderstanding could be that the pressure level that we dealt with was slightly higher than the conventional range for tactile sensing pressure. However, a reasonable tactile pressure range could be realized by varying one of the MWCNT-PDMS composite traits such as the CNT content, Young’s modulus of PDMS, the thickness, or the CNT distribution. In this regard, a softer, thinner MWCNT-PDMS composite together with a different DNN architecture achieved the detection of the conventional tactile range (0~70 kPa) at each sector position, and the results are presented in the Supplementary information (S7 and S8). Consequently, the proposed approach exhibited an outstanding level of sensing versatility from a tactile range to a pain-inducing range, which is an evident distinction from existing e-skins. On the other hand, either nano- or micro-scale pattern-based e-skins would break at such a high-pressure level (≈10 MPa), but the crude MWCNT-PDMS sheet endured it. Extremely high pressures might be required for a specific application such as either military or search-rescue robot skins.

In addition, the extreme flexibility and harsh foldability of the DNN-driven MWCNT-PDMS sheet was validated in the Supplementary information (S9). In this regard, if a sign language interpreting glove was made of homogeneous MWCNT-PDMS sheets, the cost would be lowered tremendously because it would be possible to eliminate any complicated attachments on the glove surface. The simple structure of the DNN-driven MWCNT-PDMS glove contrasts sharply with the existing versions that involve complicated device structures^28–33^.

In addition to its use in e-skin and sign language interpreting glove, this novel concept could be used in many applications such as touch panels and flexible key boards. One of the most promising applications for the DNN approach would be touch panels, since this has great potential in both academia and industry. The currently available touch panel technologies never allow for pressure recognition, and only position recognition is possible^27^. Even a stepwise (two or three steps at best) classification of pressure would significantly expand the range of functions for devices. A DNN touch panel would give rise to an explosive expansion of functionality since it allows for a reliable estimation of the real value of the pressure on a specific spot, which goes beyond step classification. In addition, a multi-point touch recognition along with the exact detection of complicated pressure distribution would definitely be a possibility. However, such versatile tasks would require larger amounts of training and test data, more extensive DNNs, more computational burdens, and longer training time, which might not currently be feasible but will definitely be possible in the near future in parallel with the progress in both big-data availability and DNN technology.

The standardization of e-skin for mass production must be discussed. At this point, the mass production of any DNN-based device would be impossible because the training costs would be astronomical. The production of every single sheet would require independent training. For example, every e-skin must receive time-consuming training processes such as a huge number of repetitive loadings, touches, twisting, pulling, and so forth. Moreover, as the sensory function demands get more sophisticated, the training becomes more time consuming. As previously mentioned, to collect a sufficient amount of data for DNN training requires tremendous time and effort when hundreds of thousands of e-skins must be trained via an iterative mechanical loading process. Once a certain degree of standardization becomes available then no extra effort would be required for such an inefficient reiterative training process. The training of an e-skin would apply to all other standardized replicas. In pursuit of this goal, we have continued manufacturing a number of homogeneous MWCNT-PDMS composite sheets with extreme vigilance and testing them using the same wooden bar (not toy finger in this case). These semi-standardized sheets recorded test accuracies in the range from 66.67 to 79.16%. Although this result was disappointing, it suggests the possibility of standardization for mass production.

## Conclusions

A deep-learning technique was used to produce a 40 × 40 mm^2^ simple flat piezoresistive MWCNT-PDMS sheet that could be used as a versatile sensory device for a variety of applications such as an e-skin. As an e-skin, pressure could be instantaneously detected at the position the pressure was applied within a macro-scale area. This revolutionary approach is unprecedented since all currently available e-skins feature a complicated and device-oriented construction consisting of multi-layered complex patterns of device components and depend on high-cost manufacturing processes such as complex cast molding, ink-jet or roll-to-roll printing, vaporized deposition, and even more costly semiconductor processing, but our much simpler approach reaches the same level of functional performance.

Training and test datasets for the DNN were collected by applying repetitive pressure to selective small sectors of the virtual lattice on the MWCNT-PDMS sheet. The DNN architecture was semi-optimized via a huge number of trials from scratch. The number of probe terminals for resistance detection was minimized while keeping the test accuracy acceptable. We finally obtained a 97.22% level of test accuracy for position recognition along with a reliable pressure estimation with a 3.12% RMSE for a 6 × 6 lattice with 8 probe terminals. This performance approximated the capability of human skin.

The proposed DNN-driven e-skin sensor outperformed present sensors in terms of pressure sensitivity and spatial resolution when the number of probe terminals was set at 16. The test accuracy for position recognition was exactly 100% for all the virtual lattice designs (4 × 4, 6 × 6, and 10 × 10). A regression-typed DNN gave a brilliant spatial resolution (0.78 ± 0.44 mm) for position recognition. Also, a high precision of pressure sensing (%RMSE ~3) was secured.

It is plausible that the successful sensory performance of this MWCNT-PDMS sheet could guarantee versatility even when more complex pressure modes are operated on irregular-shaped areas. This novel concept wherein a simple material could act as a smart, complicated device via the assistance of deep learning could open new horizons for many fields of device engineering.

## Methods

### Sample preparation

A homogeneous composite material was synthesized from commercially available multi-walled carbon nanotube (MWCNT) (Carbon Nano-material Technology Co. Ltd) and polydimethylsiloxane (PDMS) (Sylgard® 184 Silicone Elastomer). The CNT was multi-walled with a diameter of 20 nm and a length of 5 μm. To construct a piezoresistive sheet (or film), 1 wt % of the CNT was mixed with liquid PDMS in a plastic cylindrical container. In order to homogeneously disperse the CNT in PDMS and avoid agglomeration, a few 10 mm alumina grinding balls were added and the container was transferred to a planetary shear mixer at a mixing speed of 400 rpm for 2 hr. A PDMS curing agent was then added at a weight ratio of 1:10 and again the container was transferred to the planetary shear mixer for 20 min. Finally, the composites were degassed inside a vacuum for 20 min to remove entrapped bubbles. Some samples with different curing agent ratios were also prepared to control the softness.

Using the Doctor’s Blade Technique, a piezoresistive CNT/PDMS composite was cast in a 40 mm × 40 mm × 5 mm acrylic mold. The CNT/PDMS composite was then solidified at 60 °C for 30 min. Molds with smaller depths (0.2, 1, and 5 mm) were used to cast thinner sheets. Several copper wires were connected to the specimen using the terminal of a MWCNT-PDMS extension at a distance of 3 mm from each MWCNT-PDMS sheet edge, and a ground was positioned at the center of the sheet. Since the number of the resistance measurement probe terminals plays a crucial role in operating a pattern-free strain sensor sheet, we varied it from 1 to 4 probes on each side.

### Sample training and test

The sample training was implemented on an Instron E3000. The entire piezoresistive sheet area was virtually divided into 6 × 6 sectors. The Instron E3000 was equipped with a cylindrical bar that had a round end with a cross-sectional diameter of 6 mm, which could be used to systematically push a selected sector area of the piezoresistive sheet. The piezoresistive sheet was placed on a movable x-y stage so that the pressing point could be changed. By repetitively pushing each sector area one by one, we collected electrical resistance data from 4-, 8-, and 16-probe terminal electrodes on the sheet edge. In parallel with the training dataset collection, a test dataset was independently collected using an artificial plastic finger, which was a bit softer than the original training stick and more closely approximated an actual human finger.

## Electronic supplementary material

SUPPLEMENTARY INFO
