# Supplementary material for: An extremely simple macroscale electronic skin realized by deep machine learning
Source: Sci Rep. 2017 Sep 11;7:11061. doi: 10.1038/s41598-017-11663-6 (PMC5593817; doi:10.1038/s41598-017-11663-6)
Supplement: Supplementary file 1 — SUPPLEMENTARY INFO [file 41598_2017_11663_MOESM1_ESM.pdf]

Supplementary Information

An extremely simple macroscale electronic skin realized by deep machine learning

Kee-Sun Sohn,[1,*] Jiyong Chung,[2] Min-Young Cho,[3] Suman Timilsina,[3] Woon Bae Park,[1] Myungho Pyo,[4] Namsoo Shin,[5] Keemin Sohn,[2,*] and Ji Sik Kim[3,*]

[1]Faculty of Nanotechnology and Advanced Materials Engineering, Sejong University, Seoul 143-747, Republic of Korea

[2]Laboratory of Big-data applications for public sector, Chung-Ang University, 221, Heukseok-dong, Dongjak-gu, Seoul 156-756, Republic of Korea

[3]School of Nano & Advanced Materials Engineering, Kyungpook National University, Kyeongbuk 742-711, Republic of Korea

[4]Department of Printed Electronics Engineering, Sunchon National University, Chonnam 540-742, Republic of Korea

[5]Deep Solution Inc., 2636, Nambusunhwan-ro, Seocho-gu, Seoul, 06738, Republic of Korea

* : To whom all correspondence should be addressed. (kssohn@sejong.ac.kr, kmsohn@cau.ac.kr, jskim@knu.ac.kr)

Table of Contents

S1. DNN architectures along with the test result for 6 X 6-lattice sheets

S1-1. DNN for position recognition

S1-2. DNN for pressure prediction

S1-3. Regression result for pressure sensing (16 features)

S2. DNN architectures for 4 X 4-lattice sheet for touch position recognition

S2-1. DNN with 16 features and 16 labels for position recognition

S2-2. DNN with 4 features and 16 labels for position recognition.

S3. DNN with 16 features and 100 labels for 10 X 10-lattice sheets

S3-1 DNN for position recognition

S3-2. DNN for pressure sensing

S3-3. Regression result for pressure prediction (16 features and 10 X 10 lattice e-skin)

S4. The real number regression of spatial coordinates (x and y) by DNN

S5. A systematic comparison between present e-skin techniques

S6. The comparison between DNN- and EIT-driven sensors

S6-1. Evaluation of test accuracy for the EIT-driven sensor based on pick-block type position recognition

S6-2. Evaluation of test accuracy for the regression-typed DNN-driven sensor

S7. The electric resistance vs. pressure curves for thick (hard) sheet and thin (soft) film

S8. DNN for tactile range pressure sensing with 8 features for 6 X 6-lattice sheets

S8-1. Resistance curves versus the displacement (pressure) for each of 36 sectors

S8-2 DNN with 8 feature and 36 labels for position recognition

S8-3. DNN with 8 features for pressure prediction

S8-4 Comparison between hard and soft e-skin

S9. Extreme flexibility and harsh foldability of the DNN-driven MWCNT-PDMS sheet

S1. DNN architectures along with the test result for 6 X 6-lattice sheets

S1-1. DNN for position recognition

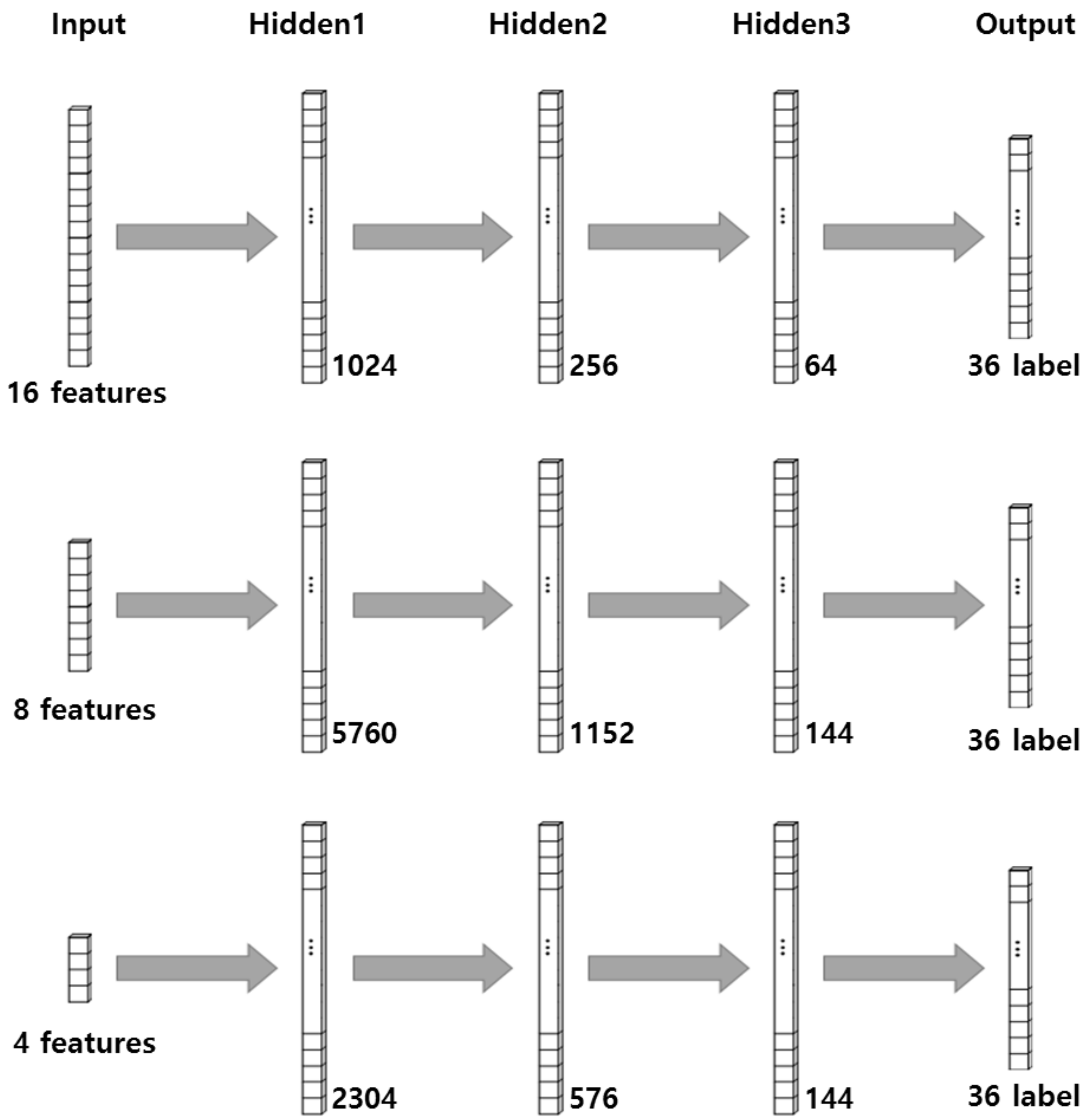

Figure S1. The DNN architectures with 4, 8, and 16 features for position recognition.

✓ 16 features

	Accuracy	Loss	Group Accuracy
Test	**99.28%**	0.0314	**72/72 = 100%**
Train	99.96%	0.0011	360/360 = 100%

✓ 8 features

	Accuracy	Loss	Group Accuracy
Test	**96.19%**	0.2773	**70/72 = 97.22%**
Train	98.97%	0.0263	360/360 = 100%

✓ 4 features

	Accuracy	Loss	Group Accuracy
Test	**51.33%**	4.7902	**36/72 = 50%**
Train	87.89%	0.3160	357/360 = 99.167%

Table S1. Detailed accuracy data for position recognition.

S1-2. DNN for pressure prediction

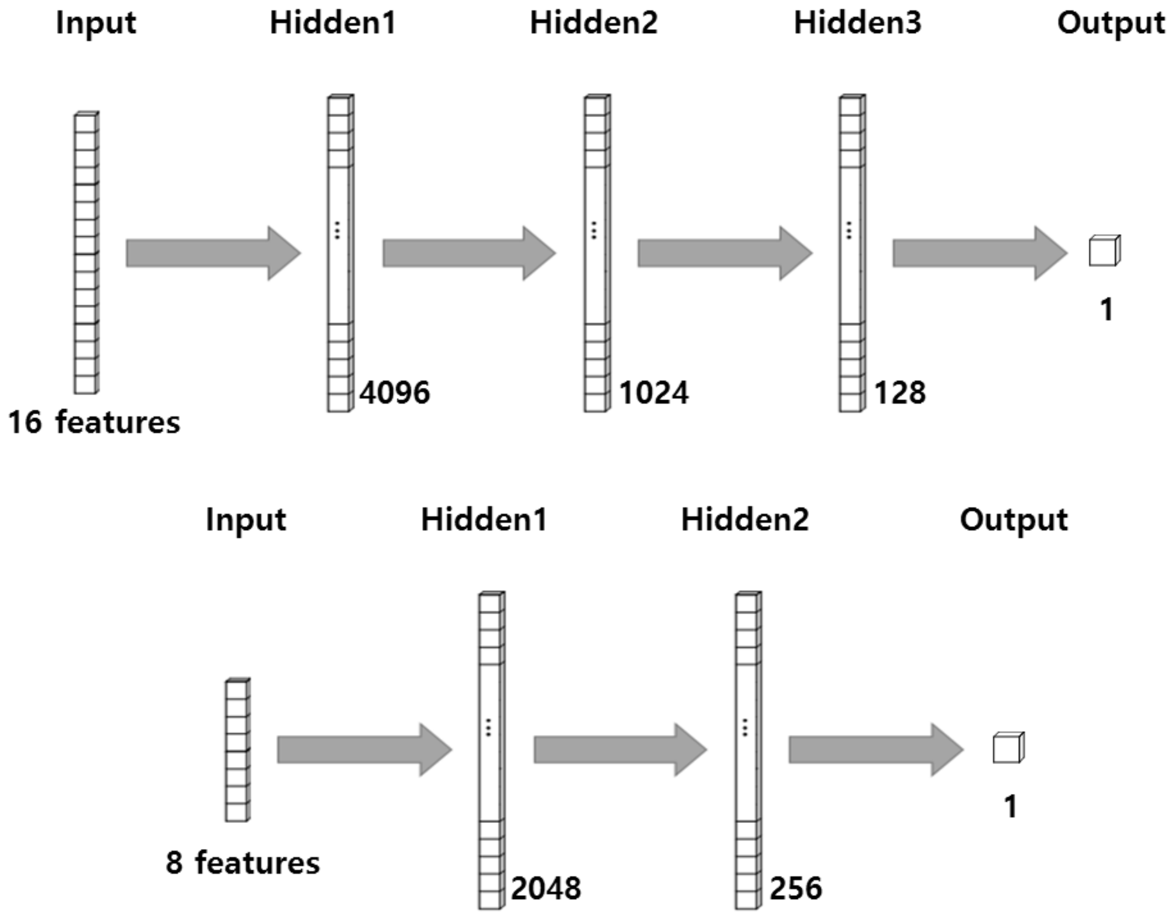

Figure S2. The DNN architectures with 8 and 16 features for pressure sensing.

	16 features	8 features
%RMSE	**3.9602**	**3.1152**
MAE	0.0533	0.0450

Table S2. Detailed test accuracy data for pressure sensing.

S1-3. Regression result for pressure sensing (16 features)

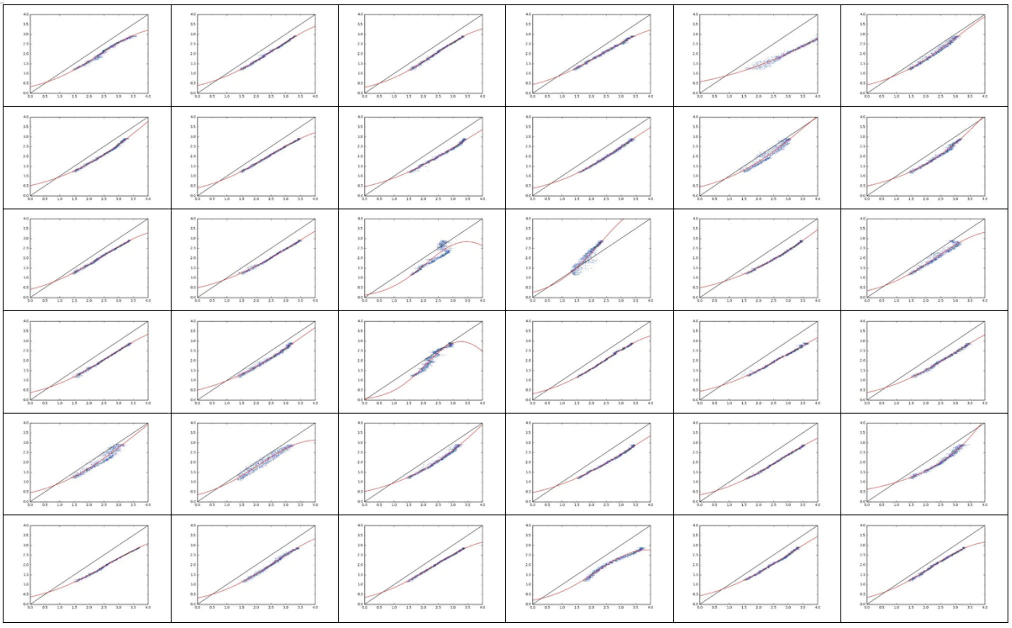

Figure S3 (a). Predicted vs measured displacement (pressure) curves for 1,000 test data point for each of the 36 sectors, a diagonal straight line stands for the optimal data status but the actual result deviated significantly from it. The red line shows the result from the polynomial function regression to compensate for the deviation.

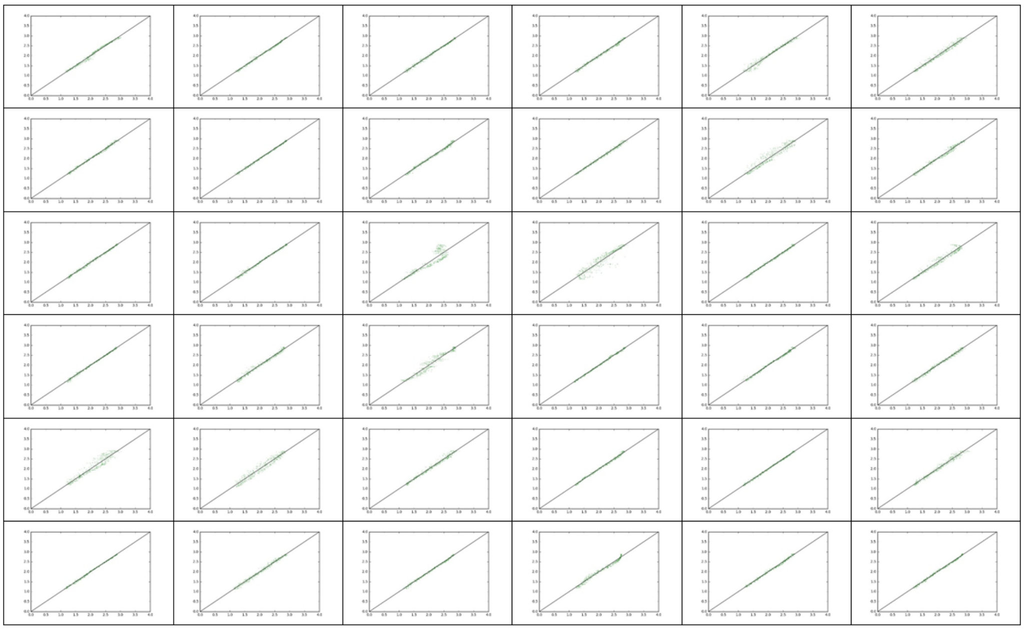

Figure S3 (b). Predicted vs measured displacement after the data transformation based on the regression result, showing the perfect coincidence between the predicted and measured displacement for every sector.

S2. DNN architectures for 4 X 4-lattice sheets for touch position recognition

S2-1. DNN with 16 features and 16 labels for position recognition.

Input data : 1x16 vector

First Layer:

Number of Nodes 1024, Activation 'ReLu', Dropout 0.3

Second Layer:

Number of Nodes 256, Activation 'ReLu', Dropout 0.3

Third Layer:

Number of Nodes 64, Activation 'ReLu', Dropout 0.3

Last Layer:

Number of Nodes 16, Activation 'Softmax'

Validation ratio : 0.25, epoch 100

Train loss: 0.0049

Train accuracy: 99.93

Test loss: 0.4845

Test accuracy: 96.57 %

Group train accuracy: 100 %

Group test accuracy: 100 %

S2-2. DNN with 4 features and 16 labels for position recognition.

Input data : 1x4 vector

First layer:

Number of Nodes 2048, Activation 'ReLu', Dropout 0.3

Second layer:

Number of Nodes 1024, Activation 'ReLu', Dropout 0.3

Third layer:

Number of Nodes 512, Activation 'ReLu', Dropout 0.3

Fourth layer:

Number of Nodes 256, Activation 'ReLu', Dropout 0.3

Fifth layer:

Number of Nodes 128, Activation 'ReLu', Dropout 0.3

Sixth layer:

Number of Nodes 64, Activation 'ReLu', Dropout 0.3

Last layer:

Number of Nodes 16, Activation 'Softmax'

Validation ratio : 0.25, epoch 1000

Train loss: 0.4771 %

Train accuracy: 80.11 %

Test loss: 2.0332 %

Test accuracy: 63.51 %

Group train accuracy: 97.5 %

Group test accuracy: 71.88 %

S3. DNN with 16 features and 100 labels for 10 X 10-lattice sheets

S3-1 DNN for position recognition

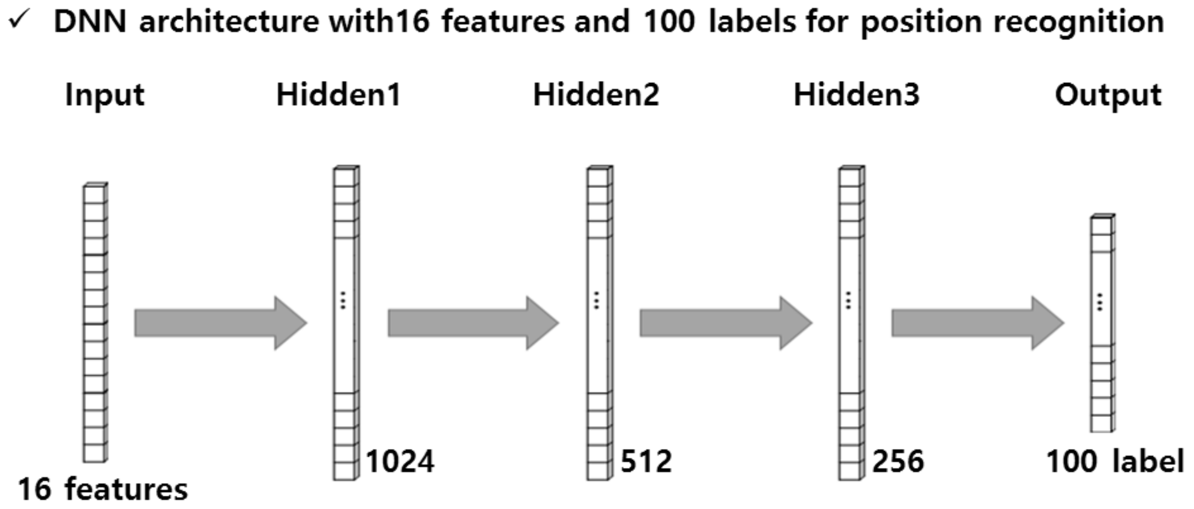

Figure S4. The DNN architecture with 16 features and 100 labels for position recognition in 10 X 10 lattice e-skin sheet with a size of 40 mm X 40 mm.

✓ **16 features to 100 labels**

	Accuracy	Loss	Group Accuracy
Test	**78.86%**	0.6070	**100/100 = 100%**
Train	80.70%	0.6166	796/800 = 99.5%

Table S3. Detailed accuracy data for position recognition.

S3-2. DNN for pressure sensing

✓ **DNN architecture with16 features for pressure sensing**

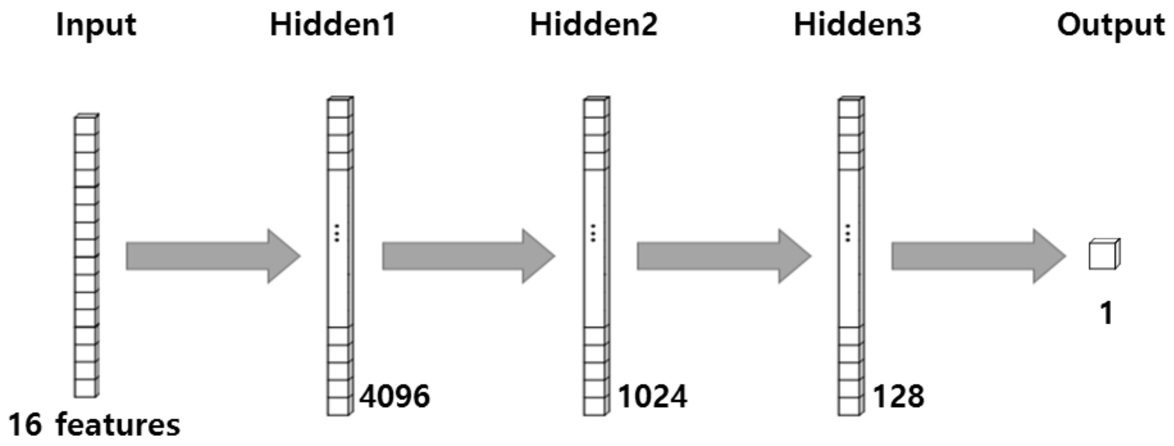

Figure S5. The DNN architecture with 16 features for pressure sensing for several representative sectors in 10 X 10 lattice e-skin sheet with a size of 40 mm X 40 mm.

(1,1)	(1,10)	(10,1)	(10,10)
(5,4)	(5,7)	(7,4)	(7,7)

Table S4. Selected sectors only were selected for use in the pressure evaluation for brevity. The sectors were selected, such that a certain symmetry pattern can be generated in the original lattice matrix. Also, the pressure evaluation on the other sectors should be definitely successful.

%RMSE	**2.8111**
MAE	0.0442

Table S5. Detailed test accuracy data for pressure sensing.

S3-3. Regression result for pressure prediction (16 features and 10 X 10 lattice e-skin)

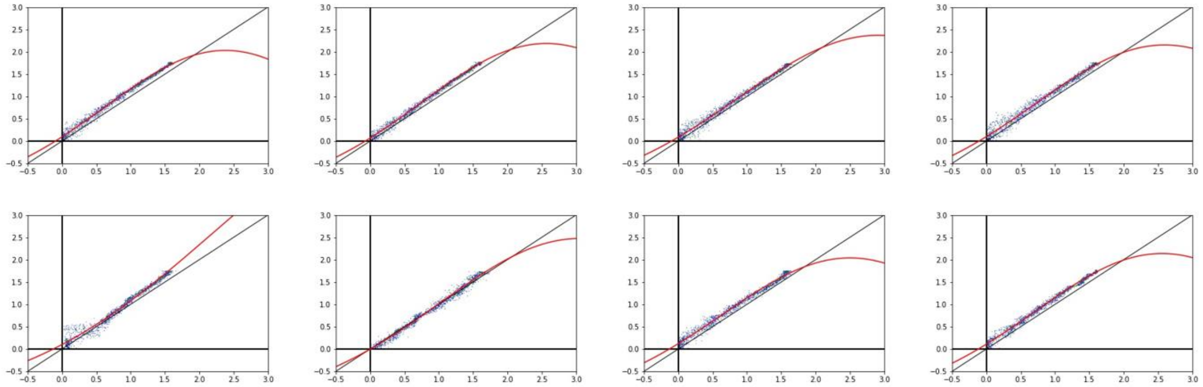

Figure S5 (a). Predicted vs measured displacement (pressure) curves for 1,000 test data point for each of the 36 sectors, a diagonal straight line stands for the optimal data status but the actual result deviated significantly from it. The red line shows the result from the polynomial function regression to compensate for the deviation.

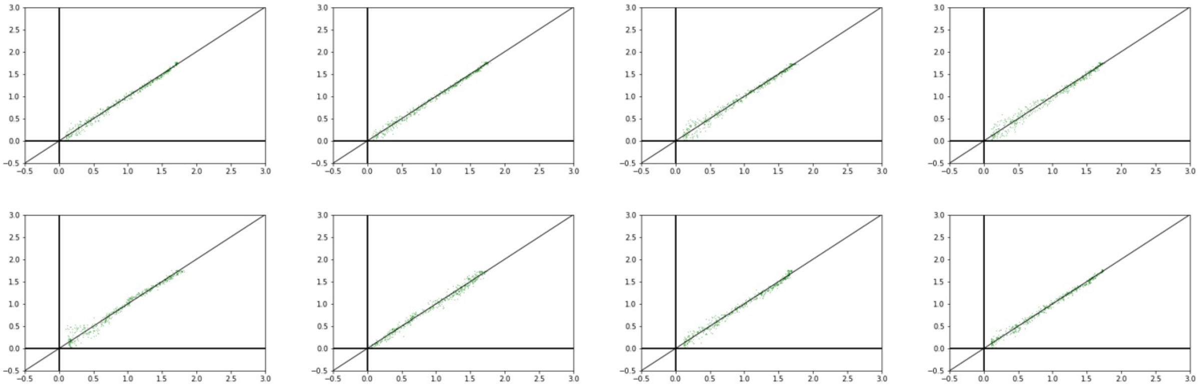

Figure S5 (b). Predicted vs measured displacement after the data transformation based on the regression result, showing the perfect coincidence between the predicted and measured displacement for every sector.

S4. The real number regression of spatial coordinates (x and y) by DNN

16 Input nodes to 2 real number output

Aside from the conventional classification DNN models dealt with in the manuscript, we developed a regression-typed DNN model to predict exact touch points rather than the identification of a sector number out of the pre-designed matrix. In this case the output is two real values designating the real coordinates (x and y) on the e-skin sheet area. **Figure S9** shows the DNN architecture. When compared to EIT-driven sensor, the resolution error much smaller. Also it should be noted that that the amount of data used for the estimation of the resolution and error values are much greater in comparison to the EIT case wherein only a touch per each position was presented.

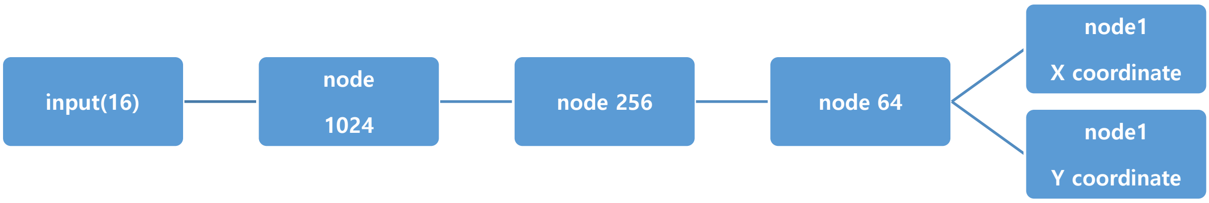

Figure S6. The regression-typed DNN architecture

	X coordinate	Y coordinate	average
%RMSE	3.24819833793	3.26769092788	3.2579446329
RMSE	0.113686941827	0.114369182476	0.114028062152
MAE	0.0848900556112	0.0943837681678	0.0896369118895
Resolution	0.78 ± 0.44 mm		

Table S6. The regression results.

S5. A systematic comparison between present e-skin techniques

		Deep machine learning + Piezoresistive (CNT+PDMS)	Patterned electronic devices for Pressure sensors	Electrical impedance tomography + Piezoresistive (CNT+silicone)
Spatial resolution	Based on Pick-block (or pixel) type	~4 mm	~2.5 mm	~5 mm
	Based on Real distance regression	0.78 ± 0.44 mm	N/A (~2.5 mm)	1.88 ± 0.96 mm
Further resolution Improvement		Re-training is only required without structural change	Structural change is required in senor	A slight structural change is required in senor
Pressure sensing	Range	Tactile to pain-inducing range<br>Hard : 0-1.2 Mpa<br>Soft : 0-70 kPa	Tactile range only<br>Soft : 0-25 kPa<br>Soft : 0-100 kPa	Tactile range only<br><br>Soft : 0-70 kPa
	Sensitivity	Excellent	Excellent	Good
Structural simplicity	Intrinsic	Simple, Pattern free	Complex, Patterned	Simple, Pattern free
	Extrinsic : No. of electrodes per unit edge length	Excellent<br>0.588 cm^{-1}	Moderate<br>2.0 cm^{-1}	Good<br>1.33 cm^{-1}
Maintenance	Flexibility	Excellent	Good	Excellent
	Foldability	Excellent	Bad	Excellent
	Curability	Easy	Impossible	Possible
Production cost		Cheap	Expensive	Cheap

Table S7. Comparison between e-skin sensors; the left column denotes our DNN approach, the data in the middle column is based on references [1~3], and the third is based on reference [15].

S6. The comparison between DNN- and EIT-driven sensors

Lee et al.* has very recently reported another type of pattern free pressure sensor based on the electrical impedance tomography (EIT). The EIT approach is a model-based numerical process and has been used for many materials for years. Although the material and the training specimen for use in their approach was similar to ours, the performance of our DNN-driven e-skin has proven to be tremendously superior to the EIT-driven e-skin in terms of the pressure sensitivity, spatial resolution, response time, pressure sensing range, and more importantly the similarity to human skin. Such superiorities, in particular, the spatial resolution and pressure sensitivity, were reasonably validated here.

We adopted a pick-block type touch position recognition to evaluate the performance of DNN-driven sensors, while the EIT-driven sensor was evaluated by the Euclidian distance between the predicted and real position of touches. Both the evaluation systems cannot be compared reasonably due to the difference in operation system. Therefore, the accuracy (or error) estimation manner was transformed to one another, such that a reasonable comparison could be available. Also, we introduced a regression-typed DNN and an independent experiment was performed for training and test for this regression-typed DNN. As a result, we achieved a real number regression of spatial coordinates (x and y) based on the Euclidian distance between the predicted and real position of touches. The test accuracy in the form of actual distance was estimated to be about 0.78 ± 0.44 mm, which is far smaller than reported values (please see subsection **S6-2**)

* Lee, H. et al. Soft Nanocomposite Based Multi-point, Multi-directional Strain Mapping Sensor Using Anisotropic Electrical Impedance Tomography. Sci. Rep. 7, 39837; doi: 10.1038/srep39837 (2017).

S6-1. Evaluation of test accuracy for the EIT-driven sensor based on pick-block type position recognition

Accuracy comparison between DNN and EIT
Based on Block (Group Data)
EIT data were converted to meet our judging criterion

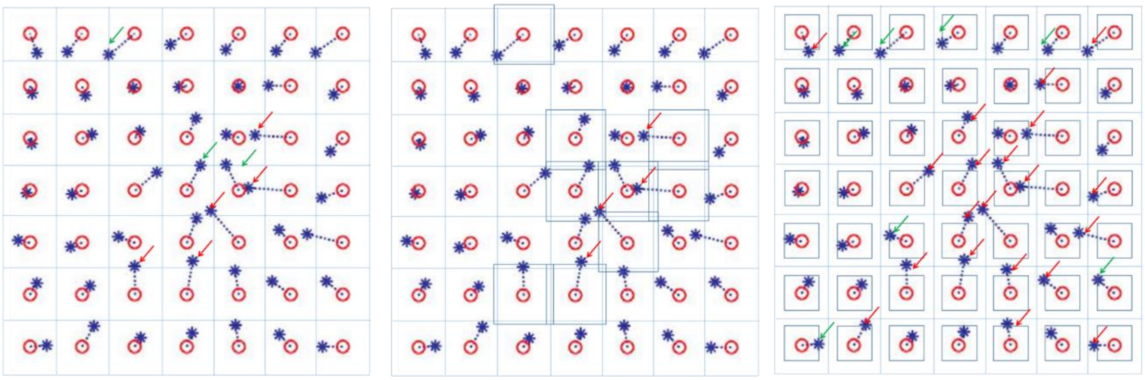

EIT e-skin Accuracy :

89.80 % (44/49) 91.84 % (45/49) 59.18 % (29/49)

Compared to

DNN e-skin Test Accuracy : 100 % (36/36 & 100/100)

Figure S7. The group-data-based test accuracy for DNN-driven sensor was consistently 100% for every lattice design (4 X 4, 6 X 6, and 10 X 10 sheets with size of 40 X 40 mm). In contrast, the accuracy for EIT-driven sensor sheet with a 7 X 7 lattice for almost the same size was reported to be 89.89. In addition, when this 7 X 7 lattice result is converted to secure an equivalent comparison condition, the accuracy was estimated to be 91.84 and 59.18 for conversions into 6 X 6 and 10 X 10 lattice cases, respectively. Slightly bigger and smaller squares than the background lattice represent the converted spatial resolution, and outliers located out of these converted resolution were counted for the accuracy calculations. Note that the DNN-driven sensor has proven to outperform the EIT-driven one in terms of the spatial resolution. Figures re-used from ref. [15].

S6-2. Evaluation of test accuracy for the regression-typed DNN-driven sensor

The test accuracy for the regression-typed DNN was estimated from the Euclidian distance between the predicted and real position of touches. The details on the regression-typed DNN architecture was well described in the section S**4**.

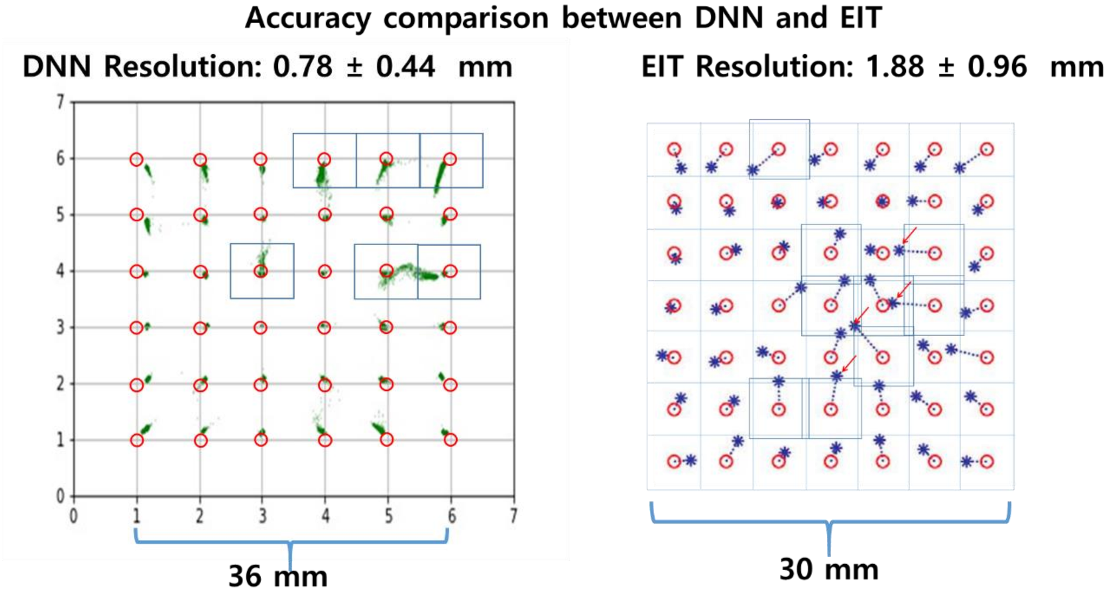

Figure S8. The test result from the regression-typed DNN architecture. We estimated the real spatial resolution to be 0.78 ± 0.44 mm, which is superior to that the EIT-driven sensor (1.88 ± 0.36). The amount of data used for the estimation of the resolution and error values are much greater in comparison to the EIT case wherein only a touch per each position was presented. The right side figure re-used from ref. [15].

S7. The electric resistance vs. pressure curves for thick (hard) sheet and thin (soft) film

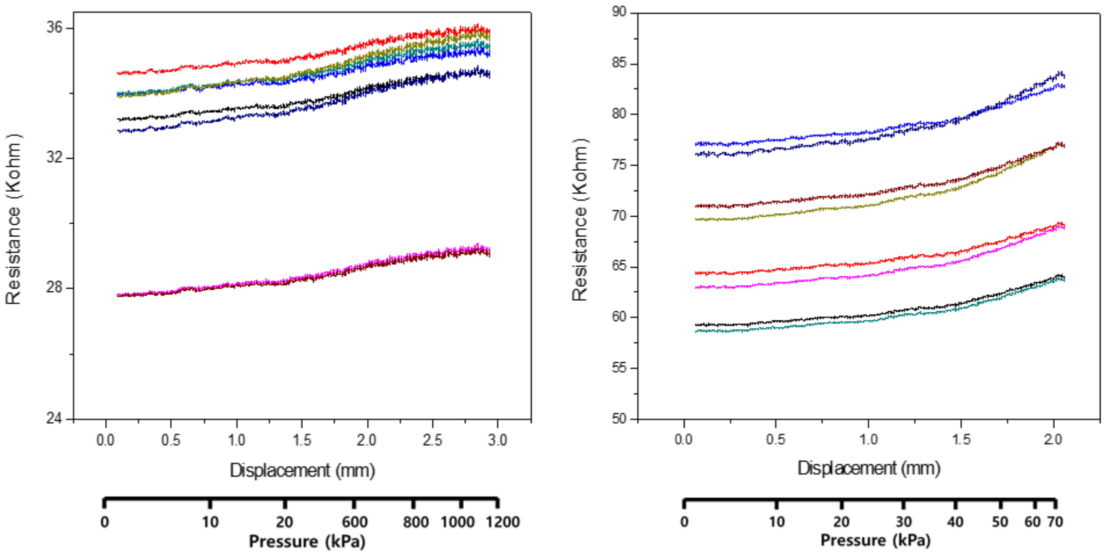

Figure S9. The eight electric resistance curves as a function of pressure at a specific sector location (4th row and 3rd column in the 6 X 6 sectoral matrix). Thick, hard sheet for high pressure evaluation (left) and thin, soft film for tactile pressure range detection (right).

S8. DNN for tactile range pressure sensing with 8 features for 6 X 6-lattice sheets

S8-1. Resistance curves versus the displacement (pressure) for each of 36 sectors

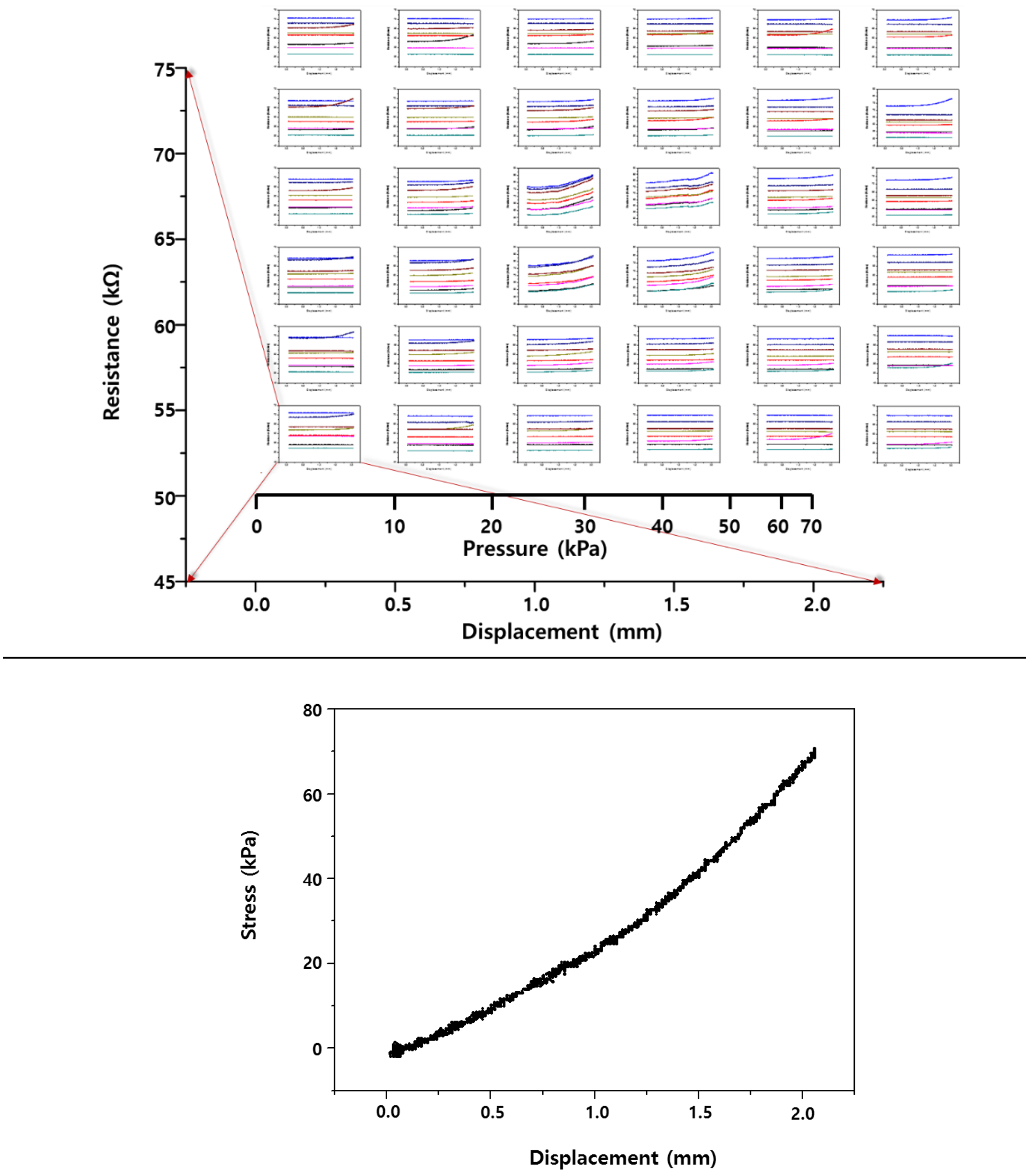

Figure S10. 8 probe terminal electrode signals versus the displacement (pressure) at 6 X 6 sector positions (upper), and a pressure versus displacement curve (bottom). **The pressure ranges from 0 to 70 kPa, which is typical tactile range.**

S8-2. DNN with 8 features and 36 labels for position recognition

Input data : 1x8 vector

First layer:

Number of Nodes 5760, Activation ‘ReLu’, Dropout 0.3

Second layer:

Number of Nodes 1152, Activation ’ReLu’, Dropout 0.3

Third layer:

Number of Nodes 144, Activation ‘ReLu’, Dropout 0.3

Last layer:

Number of Nodes 36, Activation ‘Softmax’

Validation ratio: 0.25, epoch 1000

Train loss: 0.3312 %

Train accuracy: 89.16 %

Test loss: 0.6165 %

Test accuracy: 85.54 %

Group train accuracy: 99.72 %

Group test accuracy: 100 %

S8-3. DNN with 8 features for pressure prediction

Input data: 1x8 vector.

First Layer:

Number of Nodes 2048, Activation 'ReLu', Dropout 0.3

Second layer:

Number of Nodes 256, Activation 'ReLu', Dropout 0.3

Last layer:

Number of Nodes 1, Activation 'linear'

Validation ratio: 0.1, epoch 1000

Regression model: $\ln(y) = \beta_0 + \beta_1 x + \beta_2 x^2$

%RMSE: 3.493

MAE: 0.030

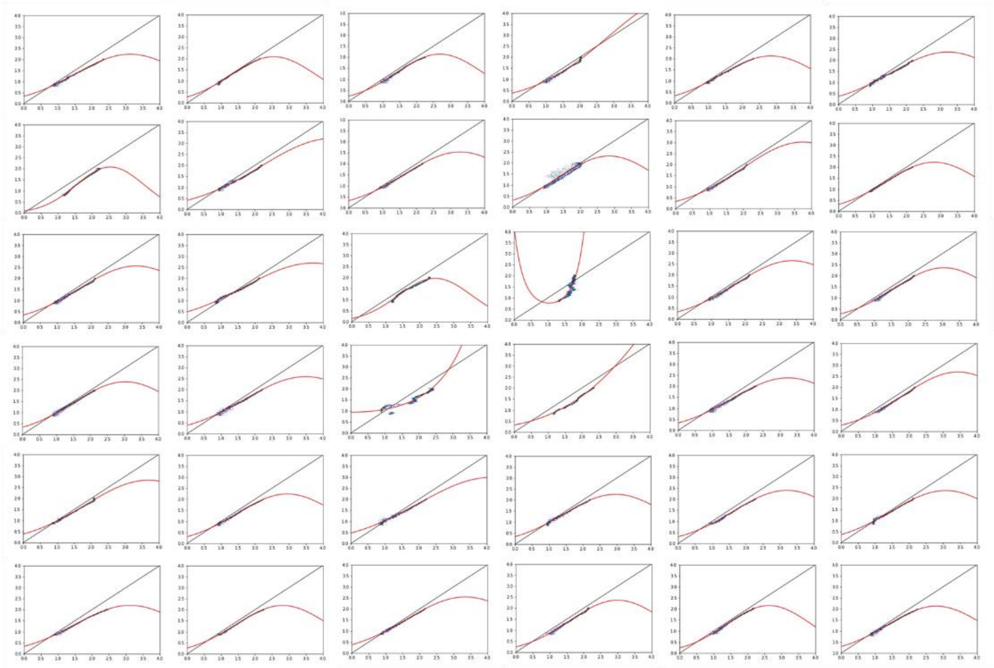

Figure S11 (a). Predicted vs measured displacement (pressure) curves for 1,000 test data point for each of the 36 sectors, a diagonal straight line stands for the optimal data status but the actual result deviated significantly from it. The red line shows the result from the polynomial function regression to compensate for the deviation.

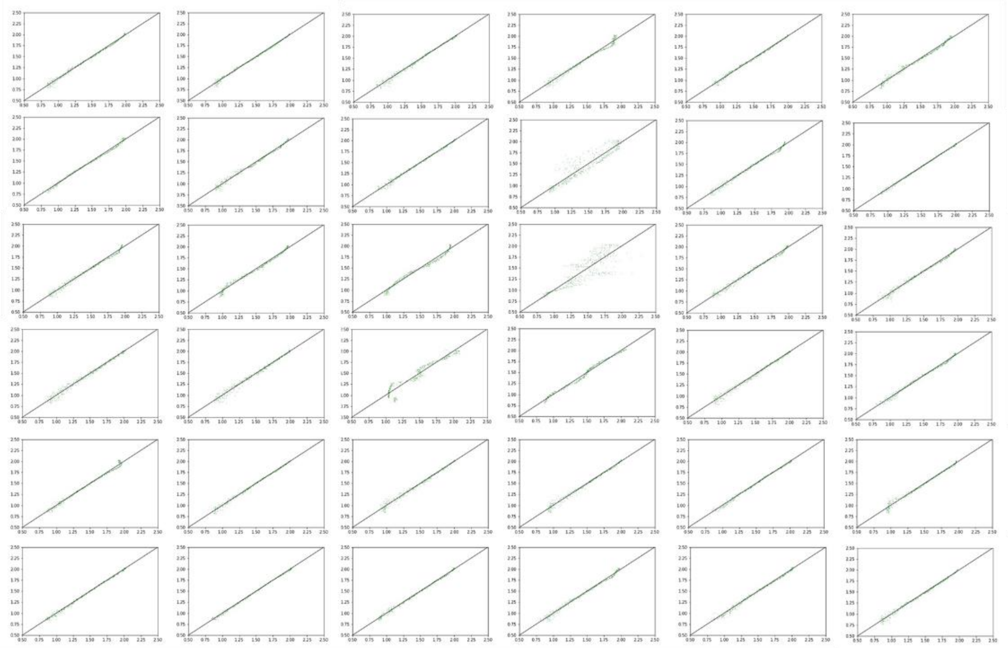

Figure S11 (b). Predicted vs measured displacement after the data transformation based on the regression result, showing the perfect coincidence between the predicted and measured displacement for every sector.

S8-4. Comparison between hard and soft e-skin

Table S8. Position recognition (8 features, 36-label classification)

Accuracy	**Thin, soft film (0.2 mm)**	**Thick, hard sheet (5 mm)**
Train loss	0.3311 %	0.0263 %
Train accuracy	89.16 %	98.97 %
Test loss	0.6165 %	0.2773 %
Test accuracy	85.54 %	96.19 %
Group-based accuracy	**Thin, soft film (0.2 mm)**	**Thick, hard sheet (5 mm)**
Train accuracy	359/360 = 99.72%	360/360 = 100%
Test accuracy	72/72 = 100%	70/72 = 97.22%

Table S9. Pressure prediction including regression

	**Thin, soft film (0.2 mm)**	**Thick, hard sheet (5 mm)**
%RMSE	3.493	3.115
MAE	0.030	0.045

S9. Extreme flexibility and harsh foldability of the DNN-driven MWCNT-PDMS sheet

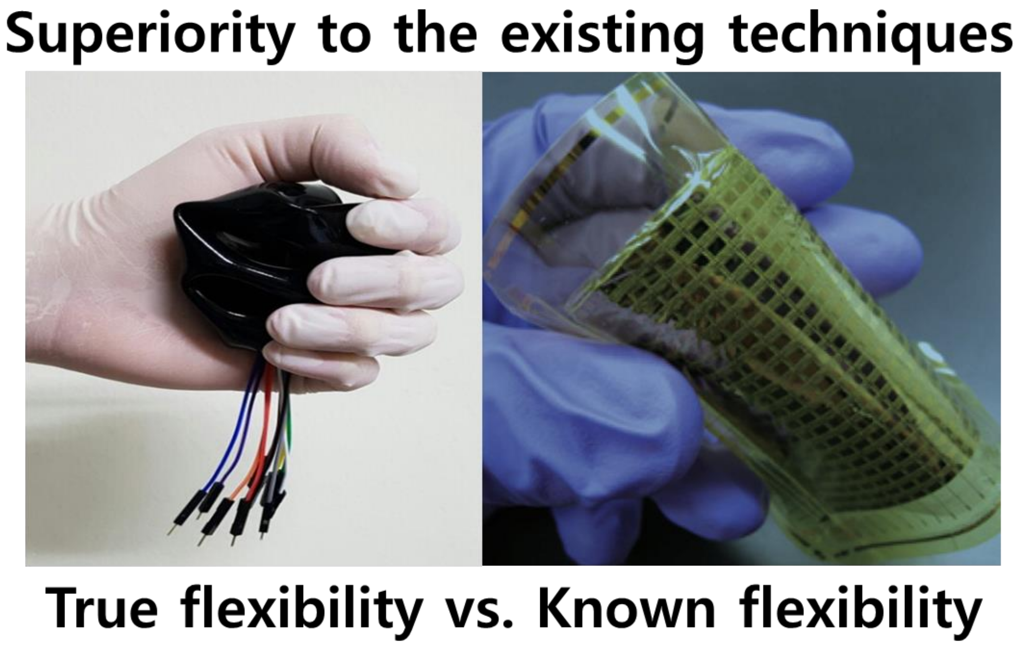

Figure S12. The DNN-driven e-skin (left side) vs. the patterned device type e-skin (right side) in terms of flexibility. In fact, the DNN-driven e-skin is now working properly even after such a harsh deformation. The right side figure was adapted with permission from ref. [2] Nature Publishing Group.
